# (*S*)-2-Carb­oxy­ethyl l-cysteinyl sulfone

**DOI:** 10.1107/S2414314624004802

**Published:** 2024-05-31

**Authors:** James K. Waters, Steven P. Kelley, Valeri V. Mossine, Thomas P. Mawhinney

**Affiliations:** a Experiment Station Chemical Laboratories, University of Missouri, Agriculture Bldg, Rm 4, Columbia, MO 65211, USA; b Department of Chemistry, University of Missouri, Columbia, MO 65211, USA; c Department of Biochemistry, University of Missouri, Columbia, MO 65211, USA; University of Kentucky, USA

**Keywords:** crystal structure, hydrogen bonding, amino acid, legumes

## Abstract

The title compound {systematic name: (2*S*)-2-aza­niumyl-3-[(2-carb­oxy­ethane)­sulfon­yl]propano­ate}, C_6_H_11_NO_6_S, forms enanti­opure crystals in the monoclinic space group *P*2_1_ and exists as a zwitterion, with a protonated α-amino group and a deprotonated α-carboxyl group. Both the carboxyl groups and the amino group are involved in an extensive multicentered inter­molecular hydrogen-bonding scheme. In the crystal, the diperiodic network of hydrogen bonds propagates parallel to (101) and involves inter­connected heterodromic *R*_4_^3^(10) rings. Electrostatic forces are major contributors to the structure energy, which was estimated by DFT calculations as *E*_total_ = −333.5 kJ mol^−1^.

## Abstract 2

The mol­ecule is a zwitterion, with a protonated α-amino group and a deprotonated α-carboxyl group. Within the crystal, the mol­ecules are linked by a system of hydrogen bonds formed by both the protonated and deprotonated carb­oxy­lic groups, and the protonated ammonium group.

## Graphical abstract

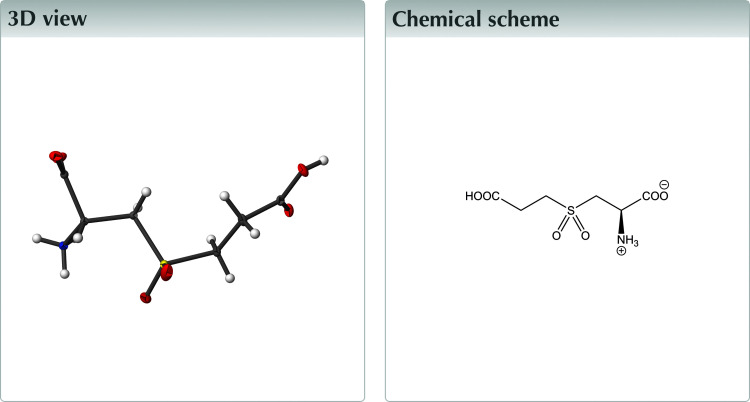

## Structure description

*S*-(2-Carb­oxy­eth­yl)-l-cysteine (CEC) and its sulfoxide (CECO) are naturally occurring, insecticidal amino acids, most often found in legumes of tropical and subtropical regions (Romeo & Simmonds, 1989 ▸; Seneviratne & Fowden, 1968 ▸). These non-proteinogenic acids have also been detected in the urine of humans exposed to dietary or occupational acryl­amide (Bull *et al.*, 2005 ▸), as well as in cysta­thio­ninuria patients (Watanabe *et al.*, 1991 ▸). Recently, we have described structures and demonstrated the protective effects of both CEC and CECO against hydroxyl free-radical induced DNA degradation (Waters *et al.*, 2022 ▸). In addition, these amino acids activated the anti­oxidant signaling pathway in renal tubular epithelial cells and protected the cells from cytotoxic CuO nanoparticles. In a continuation of our studies on anti­oxidant amino acids (Waters *et al.*, 2020 ▸, 2022 ▸; Mawhinney *et al.*, 2020 ▸), we have synthesized *S*-(2-carb­oxy­eth­yl)-l-cysteine sulfone (CECO2, **I**), an alleged metabolite of CEC, and report here its mol­ecular and crystal structures.

**Figure 1:**
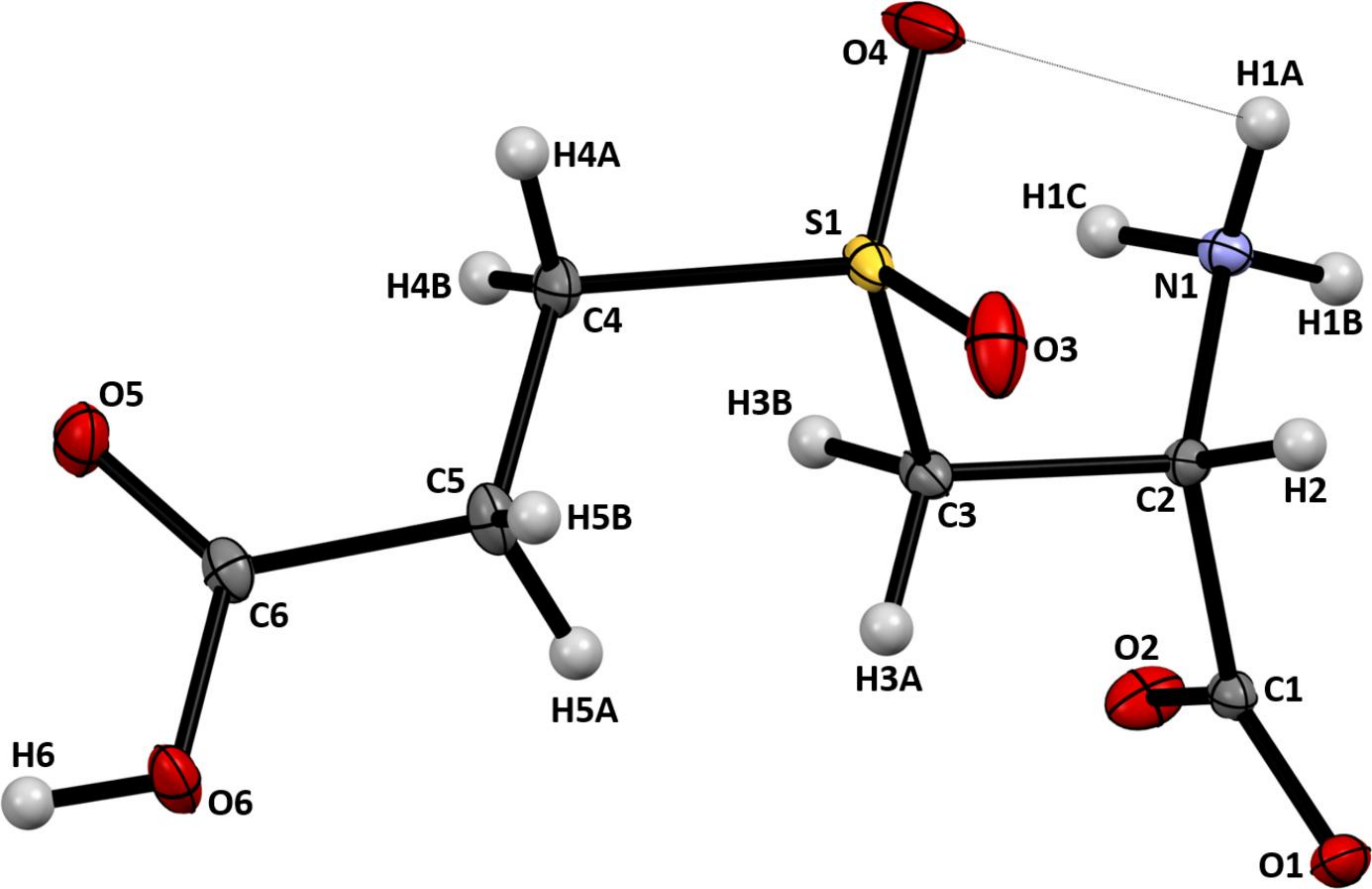
Atomic numbering and displacement ellipsoids at the 50% probability level for **I**. The intra­molecular hydrogen bond is shown as a dotted line.

Searches of SciFinder and the Cambridge Structural Database (Groom *et al.*, 2016 ▸) by both structure and chemical names revealed no previous structural description of *S*-(2-carb­oxy­eth­yl)-l-cysteine sulfone. The most closely related structures solved by diffraction methods are *S*-(2-carb­oxy­eth­yl)-l-cysteine, *S*-(2-carb­oxy­eth­yl)-l-cysteine sulfoxide (Waters *et al.*, 2022 ▸), *S*-carb­oxy­methyl-l-cysteine sulfone (CMCO2; Hubbard *et al.*, 1976 ▸), and *S*-carb­oxy­methyl-l-cysteine sulfoxide (CMCO; Staffa *et al.*, 1976 ▸; Waters *et al.*, 2020 ▸). The asymmetric unit in crystalline **I** contains one mol­ecule of the amino acid existing as a zwitterion, with a deprotonated α-carb­oxy­lic group, and protonated α-amino and ɛ-carb­oxy­lic groups, as shown in Fig.1. The aforementioned related mol­ecules uniformly adopt similar zwitterionic arrangements in their structures. All bond lengths and angles in **I** are within their expected ranges. The conformation of the cysteine moiety in **I** is close to that found in CMCO2 (CCDC #1134461, refcode CXMCYS), triclinic (4*R*)-CMCO (CCDC #2027234, refcode CMXLCS01), and may be partially stabilized in all three structures by weak intra­molecular hydrogen bonds, which exist between the sulfone/sulfoxide oxygen atom O4 and the ammonium group (Fig. 1 ▸, Table 1 ▸).

**Figure 2:**
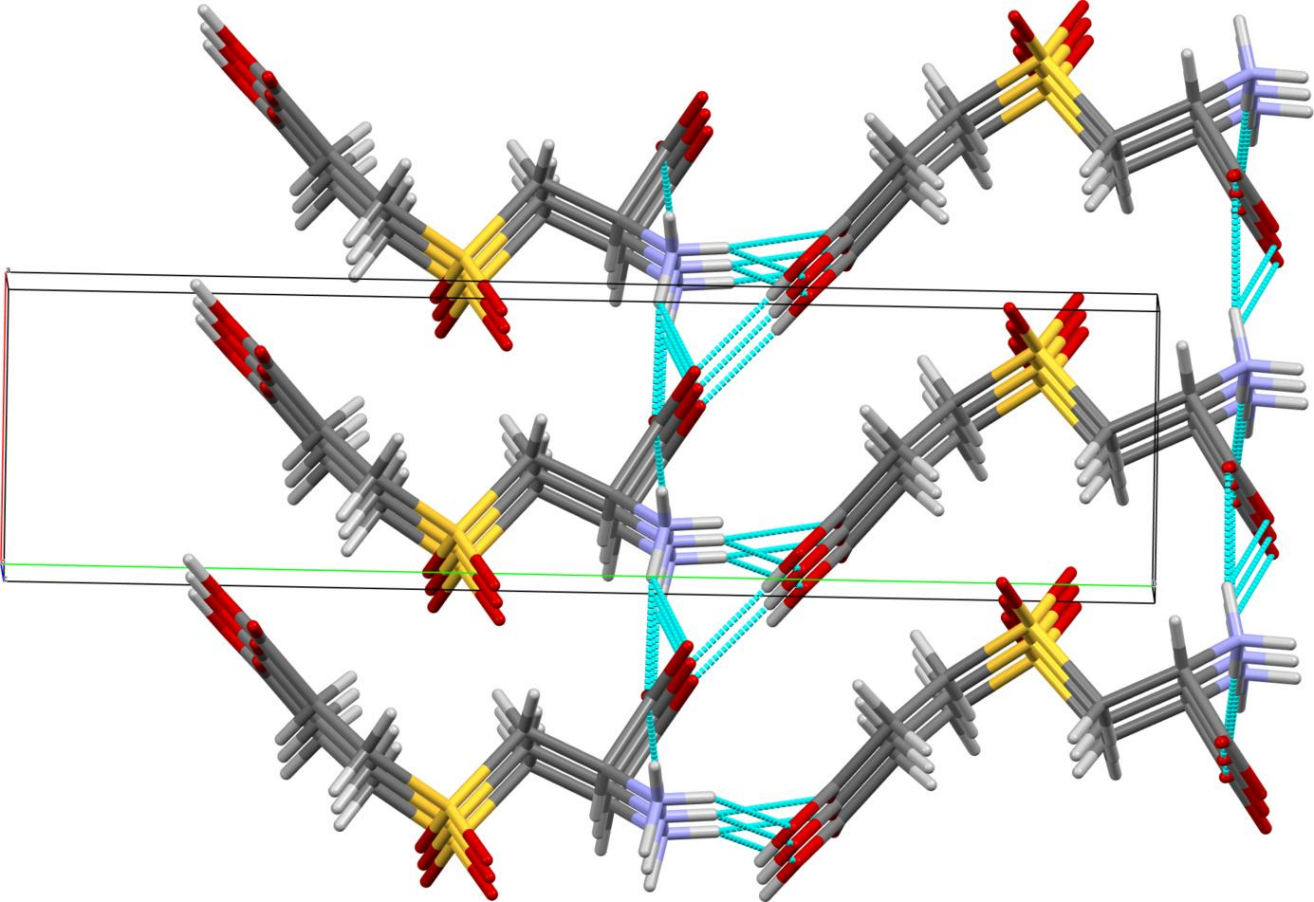
Mol­ecular packing of **I**. Inter­molecular hydrogen bonds are shown as cyan dotted lines. Crystallographic axes color codes: *a* – red; *b* – green; *c* – blue.

The crystal packing in **I** is shown in Fig. 2 ▸. The enanti­opure crystal of **I** has the symmetry of the monoclinic Sohncke space group *P*2_1_, with two mol­ecules per unit cell. Because this di­carb­oxy­lic amino acid is a heteroatom-rich, zwitterionic mol­ecule, there is an extensive inter­molecular hydrogen-bonding network, which involves all carb­oxy­lic oxygen atoms and all protons in the ammonium group, as listed in Table 1 ▸. The ammonium hydrogen atoms H1*B* and H1*A* are both involved in bifurcated hydrogen bonds. Among the oxygen atoms, the carb­oxy­lic O1 participates in multi-centered hydrogen bonding, while the sulfone O3 is the only oxygen atom not involved in heteroatom contacts. The hydrogen-bonding network topology consists of a system of heterodromic 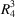 (10) rings including both α- and ɛ-carb­oxy­lic groups and the ammonium group. The rings are connected by the N1—H1*C*⋯O1 and the N1—H1*B*⋯O6 links, which propagate in the [100] and [001] directions, respectively. In addition, short C—H⋯O contacts are present in the crystal structure of **I** (Table 2 ▸).

**Figure 3:**
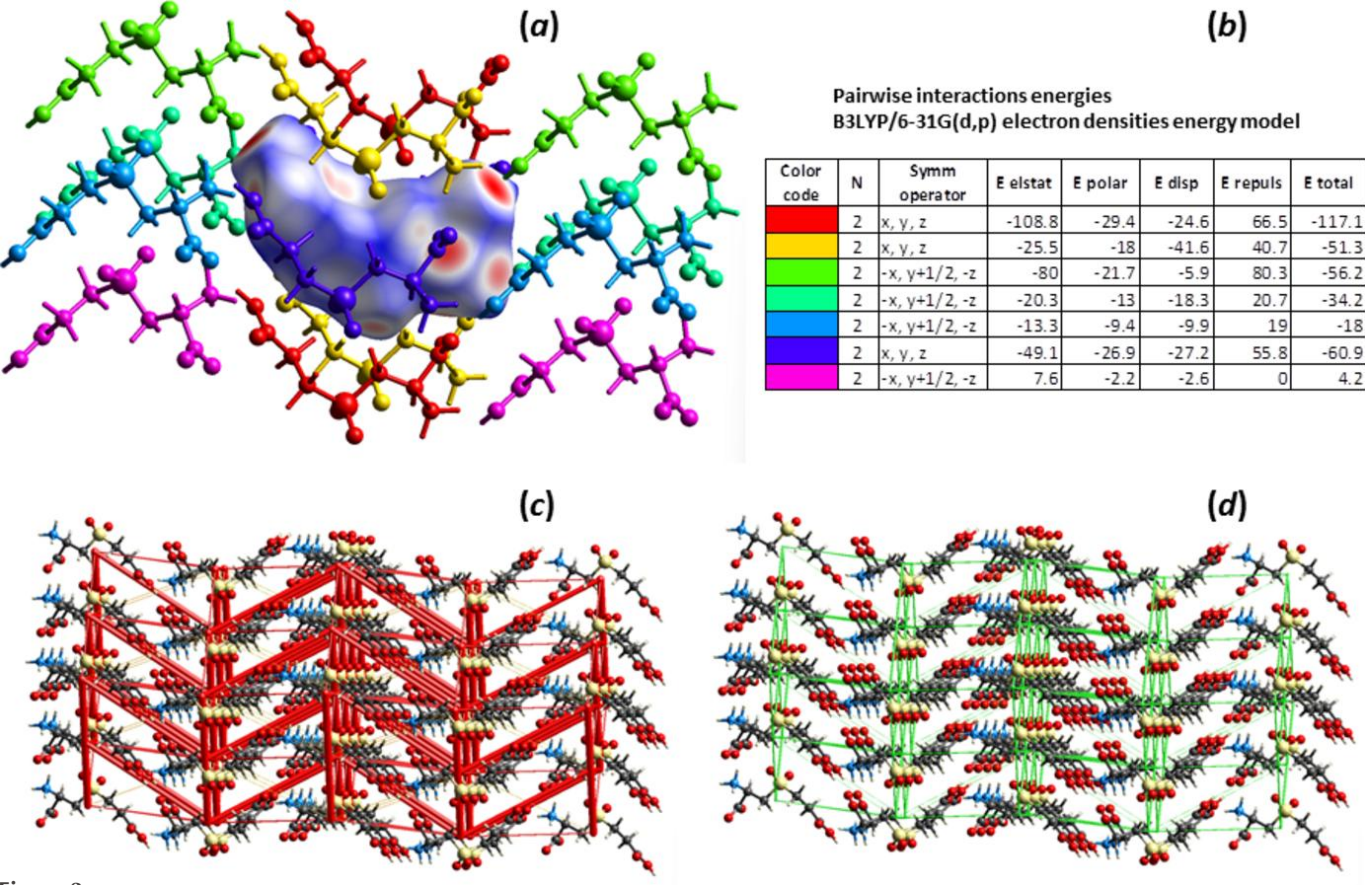
Inter­action energies in crystal structure of **I**. (*a*) A view of inter­actions between a central mol­ecule of CECO2 in crystalline **I**, shown as its Hirshfeld surface, and 14 mol­ecules that share the inter­action surfaces with the central mol­ecule. (*b*) Calculated energies (electrostatic, polarization, dispersion, repulsion, and total) of pairwise inter­actions in **I** between the central mol­ecule and those indicated by respective colors. (*c*) Energy framework for pairwise electrostatic inter­action energies in **I**. The cylinders link mol­ecular centroids, and the cylinder thickness is proportional to the magnitude of the energies, such as those shown in (*b*). For clarity, the cylinders corresponding to energies <5 kJ mol^−1^ are not shown. (*d*) The pairwise dispersion energy framework in **I**.

To account for all inter­actions involved in the crystal structure of **I**, we have performed DFT calculations, at the B3LYP/6–31 G(d,p) theory level (Thomas *et al.*, 2018 ▸; Mackenzie *et al.*, 2017 ▸), of the electrostatic, dispersion, polarization, and repulsion energies for the structure. The mol­ecular modeling calculations show that electrostatic forces arising from multiple heteroatom contacts between CECO2 mol­ecules are the main contributors to the crystal packing energies (Fig. 3 ▸, Table 3 ▸). The spatial distribution of the energetically most significant inter­actions is also illustrated in Fig. 3 ▸. As was previously noted (Waters *et al.*, 2022 ▸), there is a relatively large difference in total structural energy estimated for CECO epimers, due to a more extensive hydrogen-bonding network found in the crystal structure of the (4*R*)-epimer, as compared to that of the (4*S*)-epimer (Table 3 ▸). Both electrostatic and total energies estimated for **I** are close to those calculated for both (4*R*)-CECO and more compact mol­ecules CMCO and CMCO2.

## Synthesis and crystallization

Compound **I** was synthesized by performic acid oxidation of *S*-(2-carb­oxy­eth­yl)-l-cysteine. CEC was prepared as reported earlier (Waters *et al.*, 2022 ▸). Performic acid was made fresh by adding 10 ml of 30% hydrogen peroxide to 90 ml of 98% formic acid. Then 20 g (0.104 moles) of CEC were dissolved in 100 ml of cold performic acid and left overnight in an ice bath. The reaction was monitored using an amino acid analyser (Hitachi L8900). Upon reaction completeness, the performic acid solution was left at room temperature for 1 h, cooled down to −80°C, and then the excess of performic acid was removed by vacuum freeze drying at −50°C. The residue was recrystallized from water to afford chromatographically pure **I** as colorless plates. [α]_D_^23^ +10.9° (*c* 1, 0.2 N HCl). Elemental analysis: calculated for C_6_H_11_NO_6_S: N, 6.22%. Found: N, 6.17%. Exact mass of the [*M*+H]^+^ ion. Calculated for C_6_H_12_NO_4_S: *m*/*z* 226.02. Found: *m*/*z* 226.00.

## Refinement

Crystal data, data collection and structure refinement details are summarized in Table 4 ▸. Enanti­opurity of the crystal was established on the basis of Flack absolute structure parameter determined [−0.001 (11) for 1277 quotients (Parsons *et al.*, 2013 ▸)].

## Supplementary Material

Crystal structure: contains datablock(s) I. DOI: 10.1107/S2414314624004802/pk4044sup1.cif

Structure factors: contains datablock(s) I. DOI: 10.1107/S2414314624004802/pk4044Isup2.hkl

Supporting information file. DOI: 10.1107/S2414314624004802/pk4044Isup3.cml

CCDC reference: 1936514

Additional supporting information:  crystallographic information; 3D view; checkCIF report

## Figures and Tables

**Table 1:** Hydrogen-bond geometry (Å, °)

*D*—H⋯*A*	*D*—H	H⋯*A*	*D*⋯*A*	*D*—H⋯*A*
N1—H1*A*⋯O1^i^	0.88 (3)	1.95 (3)	2.8113 (17)	168 (2)
N1—H1*A*⋯O4	0.88 (3)	2.61 (2)	3.0239 (17)	110 (2)
N1—H1*B*⋯O5^ii^	0.86 (3)	2.22 (2)	2.8860 (16)	134 (2)
N1—H1*B*⋯O6^iii^	0.86 (3)	2.31 (2)	2.9869 (15)	136 (2)
N1—H1*C*⋯O1^iv^	0.85 (3)	1.98 (3)	2.8238 (17)	173 (2)
O6—H6⋯O2^v^	0.83 (3)	1.75 (3)	2.5436 (15)	159 (2)

Symmetry codes: (i) 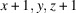 ; (ii) 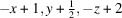 ; (iii) 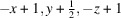 ; (iv) 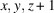 ; (v) 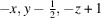 .

**Table 2:** Additional *D*—H⋯*A* contacts (Å, °)

*D*—H⋯*A*	*D*—H	H⋯*A*	*D*⋯*A*	*D*—H⋯*A*
C2—H2⋯O2^vi^	0.96 (2)	2.55 (2)	3.3104 (18)	136.3 (17)
C3—H3*A*⋯O4^vii^	0.95 (2)	2.36 (2)	3.249 (2)	155 (2)
C3—H3*B*⋯O1^iv^	0.97 (3)	2.58 (2)	3.3688 (18)	138.3 (18)
C5—H5*A*⋯O3^viii^	0.95 (2)	2.58 (2)	2.998 (2)	107.2 (16)

Symmetry codes: (vi) *x* + 1, *y*, *z*; (vii) *x* − 1, *y*, *z* − 1; (viii) *x* − 1, *y*, *z*.

**Table 3:** Calculated inter­molecular inter­action energies *E* (kJ mol^−1^) in crystalline **I** and related structures *E*_total_ = 1.057*E*_elstat_ + 0.74*E*_polar_ + 0.871*E*_energy-dispersive_ + 0.618*E*_repuls_.

Mol­ecule	*E*_electrostatic_	*E*_polar_	*E*_dispersion_	*E*_repulsion_	*E*_total_
CECO2	−289.4	−120.6	−130.1	283	−333.5
(4*R*)-CECO^*a*^	−293.6	−115.5	−130.6	308.6	−319.1
(4*S*)-CECO^*a*^	−168.6	−96.3	−92.9	187.4	−214.7
CMCO2^*b*^	−335.7	−142.4	−126	319.7	−372.6
(4*R*)-CMCO^*c*^	−336.8	−148.7	−117.3	350.2	−351.8
(4*S*)-CMCO^*c*^	−323.4	−157.7	−118.7	318.4	−365.6

Notes: (*a*) Waters *et al.* (2022 ▸); (*b*) Hubbard *et al.* (1976 ▸); (*c*) Waters *et al.* (2020 ▸).

**Table 4:** Experimental details

Crystal data	
Chemical formula	C_6_H_11_NO_6_S
*M*_r_	225.22
Crystal system, space group	Monoclinic, *P*2_1_
Temperature (K)	100
*a*, *b*, *c* (Å)	4.8838 (2), 18.3867 (7), 5.1522 (2)
β (°)	110.3246 (16)
*V* (Å^3^)	433.85 (3)
*Z*	2
Radiation type	Mo *K*α
μ (mm^−1^)	0.38
Crystal size (mm)	0.45 × 0.28 × 0.02

Data collection	
Diffractometer	Bruker APEXII area detector
Absorption correction	Multi-scan (*AXScale*; Bruker, 2014 ▸)
*T*_min_, *T*_max_	0.705, 0.746
No. of measured, independent and observed [*I* > 2σ(*I*)] reflections	15133, 2656, 2644
*R*_int_	0.017
(sin θ/λ)_max_ (Å^−1^)	0.716

Refinement	
*R*[*F* ^2^ > 2σ(*F* ^2^)], *wR*(*F* ^2^), *S*	0.019, 0.051, 1.10
No. of reflections	2656
No. of parameters	160
No. of restraints	1
H-atom treatment	Only H-atom coordinates refined
Δρ_max_, Δρ_min_ (e Å^−3^)	0.31, −0.18
Absolute structure	Flack *x* determined using 1277 quotients [(*I* ^+^)−(*I* ^−^)]/[(*I* ^+^)+(*I* ^−^)] (Parsons *et al.*, 2013 ▸)
Absolute structure parameter	−0.001 (11)

Computer programs: *APEX3* and *SAINT* (Bruker, 2014 ▸), *SHELXS* (Sheldrick, 2008 ▸), *SHELXL* (Sheldrick, 2015 ▸), *CrystalExplorer 17.5* (Mackenzie *et al.*, 2017 ▸), *Mercury* (Macrae *et al.*, 2020 ▸), *OLEX2* (Dolomanov *et al.*, 2009 ▸), and *publCIF* (Westrip, 2010 ▸).

## full crystallographic data

### Crystal data

**Table:**

C_6_H_11_NO_6_S	*F*(000) = 236
*M**_r_* = 225.22	*D*_x_ = 1.724 Mg m^−^^3^
Monoclinic, *P*2_1_	Mo *K*α radiation, λ = 0.71073 Å
*a* = 4.8838 (2) Å	Cell parameters from 9939 reflections
*b* = 18.3867 (7) Å	θ = 3.3–30.6°
*c* = 5.1522 (2) Å	µ = 0.38 mm^−^^1^
β = 110.3246 (16)°	*T* = 100 K
*V* = 433.85 (3) Å^3^	Plate, colourless
*Z* = 2	0.45 × 0.28 × 0.02 mm

### Data collection

**Table:**

Bruker APEXII area detector diffractometer	2656 independent reflections
Radiation source: Sealed Source Mo with TRIUMPH optics	2644 reflections with *I* > 2σ(*I*)
Graphite monochromator	*R*_int_ = 0.017
ω and phi scans	θ_max_ = 30.6°, θ_min_ = 2.2°
Absorption correction: multi-scan (*AXScale*; Bruker, 2014)	*h* = −6→6
*T*_min_ = 0.705, *T*_max_ = 0.746	*k* = −26→26
15133 measured reflections	*l* = −7→7

### Refinement

**Table:**

Refinement on *F*^2^	Secondary atom site location: difference Fourier map
Least-squares matrix: full	Hydrogen site location: mixed
*R*[*F*^2^ > 2σ(*F*^2^)] = 0.019	Only H-atom coordinates refined
*wR*(*F*^2^) = 0.051	*w* = 1/[σ^2^(*F*_o_^2^) + (0.0333*P*)^2^ + 0.0609*P*] where *P* = (*F*_o_^2^ + 2*F*_c_^2^)/3
*S* = 1.10	(Δ/σ)_max_ = 0.001
2656 reflections	Δρ_max_ = 0.31 e Å^−^^3^
160 parameters	Δρ_min_ = −0.18 e Å^−^^3^
1 restraint	Absolute structure: Flack *x* determined using 1277 quotients [(*I*^+^)-(*I*^-^)]/[(*I*^+^)+(*I*^-^)] (Parsons *et al.*, 2013)
Primary atom site location: structure-invariant direct methods	Absolute structure parameter: −0.001 (11)

### Special details

**Table:**

Geometry. All esds (except the esd in the dihedral angle between two l.s. planes) are estimated using the full covariance matrix. The cell esds are taken into account individually in the estimation of esds in distances, angles and torsion angles; correlations between esds in cell parameters are only used when they are defined by crystal symmetry. An approximate (isotropic) treatment of cell esds is used for estimating esds involving l.s. planes.
Refinement. Data were corrected for Lorentz, polarization, and absorption effects. Non-hydrogen atoms were refined with anisotropic thermal parameters. The hydroxyl and ammonium hydrogen atoms were located in difference Fourier maps and were allowed to refine freely. The remaining H atoms were placed at calculated positions and included in the refinement using a riding model. All hydrogen atom thermal parameters were constrained to ride on the carrier atoms (*U*_iso_(methine, methylene H) = 1.2*U*_eq_ and *U*_iso_(hydroxyl, ammonium H) = 1.5*U*_eq_).

### Fractional atomic coordinates and isotropic or equivalent isotropic displacement parameters (Å^2^)

**Table:**

	*x*	*y*	*z*	*U*_iso_*/*U*_eq_	
S1	0.85411 (6)	0.89496 (2)	0.84809 (6)	0.01103 (8)	
O6	0.1220 (2)	0.69175 (6)	0.5230 (2)	0.01356 (19)	
H6	0.006 (6)	0.6645 (14)	0.561 (5)	0.020*	
O4	1.0462 (3)	0.92863 (7)	1.0965 (3)	0.0239 (3)	
O5	0.2537 (2)	0.72208 (6)	0.9711 (2)	0.0144 (2)	
C1	0.4393 (3)	1.06770 (7)	0.3928 (3)	0.0085 (2)	
C6	0.2813 (3)	0.72813 (7)	0.7474 (3)	0.0100 (2)	
O2	0.2657 (2)	1.09831 (6)	0.4873 (2)	0.0141 (2)	
N1	0.8143 (3)	1.07575 (6)	0.8579 (2)	0.0091 (2)	
H1A	0.998 (5)	1.0653 (13)	0.951 (5)	0.014*	
H1B	0.801 (5)	1.1210 (14)	0.816 (4)	0.014*	
H1C	0.707 (5)	1.0726 (13)	0.957 (5)	0.014*	
O1	0.4180 (2)	1.06328 (6)	0.14494 (19)	0.01234 (18)	
C4	0.6697 (3)	0.82185 (7)	0.9387 (3)	0.0115 (2)	
H4A	0.818 (5)	0.7954 (13)	1.063 (4)	0.014*	
H4B	0.548 (5)	0.8430 (13)	1.023 (4)	0.014*	
C3	0.5730 (3)	0.95792 (7)	0.6791 (3)	0.0095 (2)	
C2	0.6965 (3)	1.02859 (7)	0.6080 (2)	0.0076 (2)	
H2	0.854 (5)	1.0212 (12)	0.541 (4)	0.009*	
O3	0.9803 (3)	0.87027 (6)	0.6484 (3)	0.0207 (2)	
C5	0.4975 (3)	0.77739 (7)	0.6867 (3)	0.0117 (2)	
H5A	0.395 (5)	0.8068 (13)	0.533 (4)	0.014*	
H5B	0.618 (5)	0.7471 (14)	0.621 (4)	0.014*	
H3A	0.445 (5)	0.9346 (13)	0.517 (5)	0.014*	
H3B	0.459 (5)	0.9664 (12)	0.797 (5)	0.014*	

### Atomic displacement parameters (Å^2^)

**Table:**

	*U*^11^	*U*^22^	*U*^33^	*U*^12^	*U*^13^	*U*^23^
S1	0.00737 (13)	0.00812 (13)	0.01511 (13)	−0.00088 (11)	0.00073 (9)	0.00364 (11)
O6	0.0136 (4)	0.0127 (4)	0.0158 (4)	−0.0061 (4)	0.0068 (4)	−0.0039 (3)
O4	0.0206 (6)	0.0166 (5)	0.0208 (5)	−0.0083 (4)	−0.0100 (4)	0.0045 (4)
O5	0.0184 (5)	0.0110 (5)	0.0152 (4)	−0.0032 (4)	0.0075 (4)	0.0006 (3)
C1	0.0077 (5)	0.0071 (5)	0.0096 (5)	−0.0006 (4)	0.0017 (4)	0.0009 (4)
C6	0.0097 (5)	0.0064 (5)	0.0143 (5)	0.0000 (4)	0.0047 (4)	−0.0005 (4)
O2	0.0130 (5)	0.0164 (5)	0.0130 (4)	0.0072 (4)	0.0046 (4)	0.0016 (4)
N1	0.0088 (5)	0.0086 (5)	0.0088 (4)	−0.0004 (4)	0.0015 (4)	−0.0006 (3)
O1	0.0106 (4)	0.0180 (5)	0.0079 (4)	−0.0008 (4)	0.0025 (3)	0.0012 (4)
C4	0.0118 (6)	0.0088 (5)	0.0129 (5)	−0.0024 (4)	0.0028 (4)	0.0025 (4)
C3	0.0074 (5)	0.0071 (5)	0.0122 (5)	−0.0001 (4)	0.0011 (4)	0.0008 (4)
C2	0.0079 (5)	0.0066 (5)	0.0077 (4)	0.0002 (4)	0.0019 (4)	0.0000 (4)
O3	0.0186 (5)	0.0143 (5)	0.0356 (6)	0.0035 (4)	0.0177 (5)	0.0059 (4)
C5	0.0119 (5)	0.0093 (5)	0.0157 (5)	−0.0025 (5)	0.0072 (4)	−0.0016 (4)

### Geometric parameters (Å, º)

**Table:**

S1—O4	1.4380 (12)	C3—C2	1.5293 (18)
S1—C4	1.7686 (13)	O6—H6	0.83 (3)
S1—C3	1.7756 (13)	N1—H1A	0.88 (3)
S1—O3	1.4440 (12)	N1—H1B	0.86 (3)
O6—C6	1.3285 (16)	N1—H1C	0.85 (3)
O5—C6	1.2107 (16)	C2—H2	0.96 (2)
C1—O2	1.2489 (16)	C3—H3A	0.95 (2)
C1—O1	1.2471 (15)	C3—H3B	0.97 (3)
C1—C2	1.5353 (18)	C4—H4A	0.92 (2)
C6—C5	1.5038 (19)	C4—H4B	0.93 (2)
N1—C2	1.4919 (16)	C5—H5A	0.95 (2)
C4—C5	1.5174 (19)	C5—H5B	0.95 (2)
			
O4—S1—C4	109.09 (7)	H1A—N1—H1B	109 (2)
O4—S1—C3	108.07 (7)	H1A—N1—H1C	113 (2)
O4—S1—O3	117.48 (9)	H1B—N1—H1C	102 (2)
C4—S1—C3	104.31 (6)	N1—C2—H2	106.1 (13)
O3—S1—C4	109.37 (7)	C1—C2—H2	111.4 (12)
O3—S1—C3	107.71 (7)	C3—C2—H2	113.5 (13)
O2—C1—C2	115.16 (11)	S1—C3—H3A	107.2 (15)
O1—C1—O2	127.01 (13)	S1—C3—H3B	108.2 (14)
O1—C1—C2	117.75 (12)	C2—C3—H3A	111.4 (15)
O6—C6—C5	111.32 (11)	C2—C3—H3B	112.0 (13)
O5—C6—O6	123.82 (13)	H3A—C3—H3B	106 (2)
O5—C6—C5	124.85 (12)	S1—C4—H4A	103.6 (15)
C5—C4—S1	111.44 (9)	S1—C4—H4B	105.7 (15)
C2—C3—S1	111.67 (9)	C5—C4—H4A	112.3 (14)
N1—C2—C1	108.94 (10)	C5—C4—H4B	111.7 (14)
N1—C2—C3	110.69 (10)	H4A—C4—H4B	111.7 (19)
C3—C2—C1	106.21 (10)	C4—C5—H5A	112.5 (14)
C6—C5—C4	111.54 (11)	C4—C5—H5B	113.0 (14)
C6—O6—H6	109.9 (17)	C6—C5—H5A	108.2 (15)
C2—N1—H1A	111.0 (16)	C6—C5—H5B	106.7 (15)
C2—N1—H1B	112.0 (13)	H5A—C5—H5B	104.4 (19)
C2—N1—H1C	110.2 (16)		
			
O3—S1—C3—C2	−69.00 (11)	O2—C1—C2—H2	−159.7 (14)
O4—S1—C3—C2	58.88 (12)	C1—C2—N1—H1A	−155.6 (17)
C4—S1—C3—C2	174.85 (9)	C1—C2—N1—H1B	−33.7 (18)
O3—S1—C4—C5	−41.94 (13)	C1—C2—N1—H1C	79.2 (18)
O4—S1—C4—C5	−171.68 (11)	C3—C2—N1—H1A	88.0 (17)
C3—S1—C4—C5	73.05 (11)	C3—C2—N1—H1B	−150.1 (18)
O1—C1—C2—N1	139.90 (13)	C3—C2—N1—H1C	−37.2 (18)
O1—C1—C2—C3	−100.88 (14)	H2—C2—N1—H1A	−36 (2)
O2—C1—C2—N1	−43.00 (16)	H2—C2—N1—H1B	86 (2)
O2—C1—C2—C3	76.23 (14)	H2—C2—N1—H1C	−161 (2)
N1—C2—C3—S1	−78.37 (13)	N1—C2—C3—H3A	161.7 (17)
C1—C2—C3—S1	163.54 (9)	N1—C2—C3—H3B	43.1 (16)
S1—C4—C5—C6	−165.17 (10)	C1—C2—C3—H3A	43.7 (17)
C4—C5—C6—O5	−2.7 (2)	C1—C2—C3—H3B	−75.0 (16)
C4—C5—C6—O6	177.18 (12)	H2—C2—C3—S1	40.8 (13)
O3—S1—C3—H3A	53.3 (16)	H2—C2—C3—H3A	−79 (2)
O3—S1—C3—H3B	167.4 (15)	H2—C2—C3—H3B	162 (2)
O4—S1—C3—H3A	−178.8 (16)	S1—C4—C5—H5A	−43.3 (17)
O4—S1—C3—H3B	−64.8 (15)	S1—C4—C5—H5B	74.7 (16)
C4—S1—C3—H3A	−62.8 (16)	H4A—C4—C5—C6	79.1 (16)
C4—S1—C3—H3B	51.2 (15)	H4A—C4—C5—H5A	−159 (2)
O3—S1—C4—H4A	79.0 (14)	H4A—C4—C5—H5B	−41 (2)
O3—S1—C4—H4B	−163.4 (13)	H4B—C4—C5—C6	−47.2 (15)
O4—S1—C4—H4A	−50.8 (14)	H4B—C4—C5—H5A	75 (2)
O4—S1—C4—H4B	66.8 (13)	H4B—C4—C5—H5B	−167 (2)
C3—S1—C4—H4A	−166.1 (14)	H5A—C5—C6—O5	−127.0 (14)
C3—S1—C4—H4B	−48.4 (13)	H5A—C5—C6—O6	52.9 (14)
H6—O6—C6—O5	−1 (2)	H5B—C5—C6—O5	121.2 (13)
H6—O6—C6—C5	179 (2)	H5B—C5—C6—O6	−59.0 (13)
O1—C1—C2—H2	23.2 (14)		

### Hydrogen-bond geometry (Å, º)

**Table:**

*D*—H···*A*	*D*—H	H···*A*	*D*···*A*	*D*—H···*A*
N1—H1*A*···O1^i^	0.88 (3)	1.95 (3)	2.8113 (17)	168 (2)
N1—H1*A*···O4	0.88 (3)	2.61 (2)	3.0239 (17)	110 (2)
N1—H1*B*···O5^ii^	0.86 (3)	2.22 (2)	2.8860 (16)	134 (2)
N1—H1*B*···O6^iii^	0.86 (3)	2.31 (2)	2.9869 (15)	136 (2)
N1—H1*C*···O1^iv^	0.85 (3)	1.98 (3)	2.8238 (17)	173 (2)
O6—H6···O2^v^	0.83 (3)	1.75 (3)	2.5436 (15)	159 (2)

Symmetry codes: (i) *x*+1, *y*, *z*+1; (ii) −*x*+1, *y*+1/2, −*z*+2; (iii) −*x*+1, *y*+1/2, −*z*+1; (iv) *x*, *y*, *z*+1; (v) −*x*, *y*−1/2, −*z*+1.
